# A novel 2-oxopyrrolidine derivative (LN-53) efficiently induces Nrf-2 signaling pathway activation in human epidermal keratinocytes

**DOI:** 10.1007/s43440-025-00757-y

**Published:** 2025-06-24

**Authors:** Basak Ezgi Sarac, Laura Nissim, Dilara Karaguzel, Gokhan Arik, Shirin Kahremany, Edward E. Korshin, Arie Gruzman, Cagatay Karaaslan

**Affiliations:** 1 Department of Biology, Molecular Biology Section, Faculty of Science, Hacettepe University, Beytepe Campus, Cankaya/Ankara, 06800 Turkey; 2 Department of Chemistry, Faculty of Exact Sciences, Bar-Ilan University, Ramat-Gan, 5290002 Israel

**Keywords:** Nrf-2, Keap-1, Oxidative stress, Antioxidants, Keratinocytes, 2-oxopyrrolidine scaffold

## Abstract

**Background:**

The skin is a pivotal organ that serves as a physical barrier, protecting the body from harmful substances such as pathogens, allergens, and other environmental irritants. Chronic inflammation in the skin, along with the anthropogenic effects, can cause reactive oxygen species (ROS) overproduction. Prolonged exposure to elevated ROS levels and inadequate antioxidant defenses in the skin can contribute to the onset of various skin disorders. The nuclear factor erythroid 2-related factor-2 (Nrf-2) signaling pathway plays a key role in enhancing antioxidant capacity by promoting the production of antioxidant and detoxifying molecules. Consequently, pharmacological activation of the Nrf-2 pathway may help restore the oxidant-antioxidant balance, thereby improving therapeutic outcomes for chronic skin disorders. This study aimed to investigate the potential effect of novel agent: (5-((4-(4-(methoxycarbonyl)-2-oxopyrrolidin-1-yl)phenyl)carbamoyl)benzene-1,2,3-triyl triacetate (LN-53), synthesized based on the structure of previously developed by our team lead compound SK-119, on Nrf-2 signaling pathway in human epidermal keratinocytes (HEKs) at mRNA and protein level.

**Methods:**

The cytotoxicity of LN-53 was evaluated by MTT, LDH, live/dead cell staining, and caspase-3,-8,-9 multiplex activity assays. Intracellular ROS production was assessed by DCFH-DA staining. The Nrf-2 gene was silenced by transient transfection using human Nrf-2 siRNA. Nrf-2 and related factors (heme oxygenase-1 (HO-1) and NAD(P)H dehydrogenase: quinone-1 (NQO1)) were evaluated at the mRNA level by qPCR and protein level in nuclear and cytosolic fractions by Nrf-2 activation assay and Western blot. The levels of inflammatory cytokines (IL-6 and IL-8) in supernatants were determined by ELISA.

**Results:**

Our results indicate that LN-53 effectively reduces intracellular ROS production triggered by *tert*-butyl hydroperoxide (TBHP), without leading to any noticeable cell damage. It promoted the nuclear translocation of Nrf-2 and induced the production of Nrf-2, HO-1, and NQO1 at both the mRNA and protein levels. LN-53-mediated alterations in antioxidant gene expressions were blocked by Nrf-2 knockdown. LN-53 treatment also suppressed the release of IL-6 and IL-8 cytokines mediated by TBHP exposure. Additionally, novel compound LN-53 was found to be more stable than the parent compound SK-119.

**Conclusion:**

LN-53 can effectively induce antioxidant mechanisms by promoting Nrf-2 nuclear translocation and suppressing ROS production in human epidermal keratinocytes. These data may suggest that LN-53 can contribute to maintaining redox balance and homeostasis in the skin.

**Graphical Abstract:**

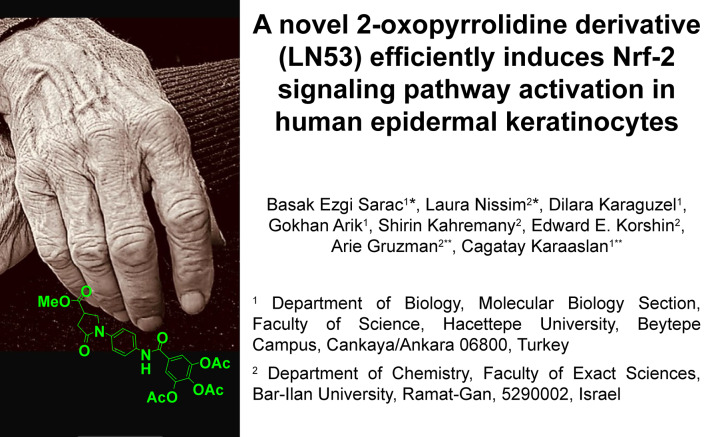

**Supplementary Information:**

The online version contains supplementary material available at 10.1007/s43440-025-00757-y.

## Introduction

 The skin is an important organ that acts as a physical barrier against external irritants, such as chemicals, environmental pollutants, allergens, and pathogens. The constant exposure to these irritants, along with the endogen pro-oxidant agents, can trigger chronic skin inflammation, which results in the overproduction of reactive oxygen species (ROS) [1]. Dermatological diseases such as atopic dermatitis and psoriasis are characterized by chronic skin inflammation and disrupted epidermal barrier [2, 3]. These skin disorders have been associated with elevated levels of oxidative stress, even at basal conditions, with aberrant ROS production during disease exacerbation. In order to maintain homeostasis, the skin cells, including keratinocytes and fibroblasts, have a variety of chemical receptors and sensors that recognize these exogenous and endogenous substances [4]. Among them, the nuclear factor erythroid-2-related factor 2 (Nrf-2)-Kelch-like erythroid cell-derived protein with cap‘n’collar homology-associated protein 1 (Keap1) signaling pathway is a crucial mechanism for maintaining redox balance within the skin [3].

Nrf-2 is a transcription factor regulating many cytoprotective and antioxidant target genes encoding more than 250 critical mediators of cellular defense functions. Nrf-2 is also involved in the regulation of various biological processes, ranging from apoptosis and inflammation to unfolded protein response [5]. Under normal physiological conditions, Nrf-2 is kept inactive by Keap1 complex (Keap1-Cul3-Rbx1) and degraded via ubiquitinoylation [6]. However, when oxidative stress within the cell increases, Keap1-Nrf-2 interaction is disrupted, and Nrf-2 is then translocated into the nucleus, thereby inducing gene expressions of antioxidant proteins such as heme oxygenase-1 (HO-1), NAD(P)H: quinone oxidoreductase 1 (NQO1), and many others, frontline players to neutralize detrimental effects of oxidants [7, 8].

It is shown that the redox imbalance in the skin can also contribute to skin inflammation in the pathogenesis of skin disorders [9]. In this context, the Nrf-2 pathway plays a pivotal role in skin homeostasis, especially in skin diseases such as atopic dermatitis and psoriasis, in which the epidermal barrier is compromised. Nrf-2 activity has been demonstrated to be closely linked to maintaining an effective epidermal barrier [10, 11]. It has also been shown that Nrf-2 activation can promote tissue-repairing mechanisms, suppress inflammasome activation, alleviate skin inflammation, as well as contribute to a functional protective barrier maintaining thiol gradient in the epidermis [3, 12]. Therefore, pharmacological activation of the Nrf-2 pathway may serve as an alternative therapeutic approach to help maintain a healthy skin condition [13]. Although the clinical trials with the topical administration of FDA-approved drugs that activate Nrf-2 like dimethyl fumarate, sulforaphane, and their derivatives, are still in progress, many ongoing studies also currently focus on designing and synthesizing new agents that will inhibit protein-protein interaction (PPI) between Nrf-2 and Keap1 complex. Such compounds might activate the Nrf-2 pathway and induce pharmacological effects in skin and in other tissues [3]. A lot of research has been conducted in recent years to develop direct inhibitors of Nrf-2/Keap1 protein/protein interaction [14]. Such molecules might activate the antioxidant response without the induction of oxidative stress and can be used for the treatment of a broad spectrum of diseases. For example, small organic molecules that can disturb the Nrf-2/Keap1 complex were reported by Narayanan D., et al. [15], by Hammoutene A [16], by Kandasamy et al. [17], Kao et al. [18], and others (for a comprehensive review, please see Gallorini et al. [19]). One such in vivo active disruptor of Nrf-2/Keap1 complex, RA839, is even a commercially available molecule [20]. Important to mention that there are many Nrf-2 non-direct activators that cause the upregulation of the Nrf-2 system not by direct disintegration between Nrf-2 and Keap1, but mostly by the changing of the redox status of SH groups in Keap1 [21, 22]. Noteworthy, the FDA approved Nrf-2 activators, such as omaveloxolone and dimethyl fumarate, for clinical use, which might cause a therapeutic effect not by direct stimulation of Nrf-2 but by recruiting alternative molecular mechanisms [23, 24]. In this context, our group also published several reports related to the development of novel Nrf-2 activators that showed high efficiency in Nrf-2 signaling activation, especially in the skin [25, 26].

In the current study, we report a newly synthesized Nrf-2 activator: 5-((4-(4-(methoxycarbonyl)-2-oxopyrrolidin-1-yl)phenyl)carbamoyl)benzene-1,2,3-triyl triacetate, LN-53 and its biological evaluation. LN-53 was designed based on the scaffold of the previous lead compound developed by our team, namely (*E*)-5-oxo-1-(4-((2,4,6-trihydroxybenzylidene)amino)phenyl) pyrrolidine-3-carboxylic acid, SK-119 that showed significant Nrf-2 activation in several in vitro (PC-12 and HaCaT cells) and ex-vivo (human skin organ culture) models. However, its chemical stability was not sufficient due to the presence of the imine bond [27]. Thus, the more stable LN-53 compound was developed, where the imine bond was replaced by a more stable amide bond. In addition, all the phenolic hydroxyls attached to the benzene ring were protected by conversion to acetates. Indeed, in this work it was shown that LN-53 is significantly more chemically stable compared to SK-119 without compromising the beneficial pharmacological properties. The ability of LN-53 to induce Nrf-2 activation and promote antioxidant mechanisms in the skin was also evaluated. Before the efficacy experiments, the possible cytotoxic effects of LN-53 on human epidermal keratinocytes (HEKs) were assayed. We also determined its capability to activate antioxidant response by inhibition of ROS production mediated by tertbutyl hydroperoxide (TBHP) in these cells. We found that LN-53 can significantly increase Nrf-2 activity at both mRNA and protein levels without exerting cytotoxicity. Finally, we observed that LN-53 can alleviate inflammation by suppressing the production of interleukins IL-6 and IL-8. We hope that such a novel Nrf-2 activator could be used for future drug development against dermatological disorders.

## Materials & methods

### Ligand preparation

The structures were prepared using the LigPrep module in the Schrodinger suite (Maestro12.7). Using an OPLS 2005 force field, the LigPrep produces energy-minimized 3D structures. For each structure, the tautomer, the correct Lewis structure, and ionization states (pH 7.0 ± 2.0) were generated, optimized, and energy minimized under default settings.

### Conformational search

Conformers of each molecule were generated in MacroModel using the OPLS_2005 force field, GB/SA water, and no cutoff for nonbonded interactions. Molecular energy minimizations were performed using the PRCG method with 5000 maximum iterations and a 0.05 gradient convergence threshold. The conformational searches were carried out by application of the Mixed torsional/Low-mode sampling method, performing automatic setup with 21 kJ/mol in the energy window for saving structure and a 0.5 Å cutoff distance for redundant conformers.

### Protein preparations and receptor grid generation

The crystal structure of Keap1 (PDB code: 2FLU) was imported into the Maestro workspace, and the multistep Protein Preparation Wizard was used to correct the protein structure, which includes the addition of H-atoms, bond order correction, and H-bond network optimization, followed by energy minimization using the OPLS 2005 force field. After preparation, the receptor grid was generated with Glide by specifying the binding site with a 3D cubic box. A box of 20 Å side lengths was placed for the enclosure. Potential energies of receptor atoms confined in this box were calculated without scaling their Van der Waals radius.

### Pharmacophore design

The structure-based pharmacophore model was generated using the LigandScout software package [28]. LigandScout is able to generate the 3D pharmacophores from structural data of macromolecule–ligand complexes. Excluded volume spheres are also included in the model based on the coordinates defined by amino acids side chain atoms to depict the inaccessible areas for any potential ligand. This pharmacophore model was used as a query in virtual screening using the Screen Library protocol, as implemented in LigandScout.

### Glide molecular docking

The molecular docking was performed with the Glide XP (Xtra Precision) protocol of the Schrodinger Suite with default settings (https://pubs.acs.org/doi/10.1021/jm0306430). Default settings were used. Docking boxes were centered on the identified site, and Glidescore was used to evaluate the resulting poses.

### Synthetic chemistry

All chemical reagents, solvents, and acids were purchased from Merck, Acros Organic, Alfa Aesar, Bio-Lab Ltd., or IU-CHEM Ltd., and all were used as received. NMR Spectra were recorded on the Bruker Avance-300 spectrometer at 25 °C in CDCl_3_. Chemical shifts are reported in ppm units (*δ*) relative to TMS (*δ*_H_ and *δ*_C_ = 0 ppm). The residual signal of under-deuterated solvent (*δ*_H_ = 7.26 ppm in CDCl_3_) in ^1^H NMR spectra, and the central peak of CDCl_3_ (*δ*_C_ = 77.00 ppm) were used as internal standards. The coupling constants (*J*), calculated according to the first-order NMR spectra, are reported in Hz. The splitting pattern abbreviations are as follows: s, singlet; d, doublet; t, triplet; q, quartet; quint, quintet; m, multiplet. High-resolution mass spectra (HRMS) were obtained on an LTQ Orbitrap XL (Thermo Scientific, Waltham, MA, USA) mass spectrometer using electrospray ionization (ESI) in a positive mode. The data were processed using mass LynX ver. 4.1 calculations and deconvolution software (Waters Corp., Milford, MA, USA). The purity of the synthesized compound LN-53 (≥ 96%) was confirmed by NMR and HPLC analysis. Analytical HPLC (Young Lin Instruments, Anyang, Korea) was performed on LUNA C_18_ analytical (5 μm, 250 × 4.6 mm) column from Phenomenex, Inc. (Torrance, CA, USA) with an increasing linear gradient of CH_3_CN in H_2_O. Analytical TLC was carried out on pre-coated silica gel 60 F_254_ (Merck) sheets using UV absorption and iodine physical adsorption for visualization. The melting point of LN-53 was measured with a Fisher-Johns melting point apparatus (Waltham, MA, USA). Column flash chromatography (FC) was performed on Silica Flash^®^ P60 silica gel (40–63 μm, 230–400 mesh ASTM) from Silicycle Inc. (Canada). All the reactions were carried out under an atmosphere of dry nitrogen in oven-dried (150 ^o^C) glassware.

**Synthesis of 5-((4-(4-(methoxycarbonyl)-2-oxopyrrolidin-1-yl)phenyl)carbamoyl)benzene-1**,**2**,**3-triyl triacetate (LN-53). A) (**To a solution of gallic acid (1.0 eq) in acetic anhydride (5.0 eq), a catalytic amount of concentrated sulfuric acid (0.2 eq) was added dropwise under stirring. Then, the reaction mixture was refluxed for 4 h. After completion (monitored by TLC), the reaction was cooled to room temperature (r.t.) and poured onto a crushed ice. The resulting precipitate was collected by filtration, washed with cold water, and dried under vacuum to give the triacetylated gallic acid (**2**). **B)** Triacetylated gallic acid (**2**) was dissolved in dry toluene (30 ml), and thionyl chloride (3.0 eq) was added dropwise in the presence of a catalytic amount of dimethylformamide (DMF). The mixture was heated under reflux for 3 h. After the evolution of gas ceased, the solvent was evaporated under vacuum to afford a crude triacetylated gallic acid chloroanhydride. (**3**). **C) (** To a stirred solution of methyl 1-(4-aminophenyl)-5-oxopyrrolidine-3-carboxylate (1.0 eq) in dry dichloromethane (DCM, 10 mL), triethylamine (2.0 eq) and dimethylaminopyridine (DMAP, 0.1 eq) were added under nitrogen atmosphere. A solution of a crude acyl chloride **3) **(from step b (1.1 eq) in DCM was added dropwise at r.t. The reaction mixture was stirred at r.t. for 12 h under dry nitrogen. Upon completion of the reaction (TLC-monitoring), the reaction mixture was washed sequentially with 1 M HCl, saturated NaHCO₃, and brine, dried over Na₂SO₄, filtered, and evaporated under vacuum. The crude product was purified by FC (elution with hexane/EtOAc 1:1) to afford **LN-53** as a yellowish solid. Yield: 42%; R_***f***_ = 0.56 (50% EtOAc in hexane); m.p. = 125 °C.

^1^H NMR (300 MHz, CDCl_3_): *δ* 2.27 (s, 6 H), 2.29 (s, 3 H), 2.86 (q, *J* = 9.7, 3.1 Hz, 2 H), 3.35 (quint, *J* = 8.7, 7.3 Hz, 1H), 3.77 (s, 3 H), 4.05(m, 2 H), 7.52 (q, *J* = 14.8, 8.5 Hz, 2 H), 7.64 (s, 2 H), 8.36 (s, 1H, NH). ^13^C NMR (75 MHz, CDCl_3_): *δ* 20.11, 20.53, 35.37, 35.81, 50.61, 52.54, 120.04, 121.09, 133.2, 137.9, 141.09, 143.76, 149.35, 163.01, 166.46, 167.61, 171.61, 172.80. HRMS (ESI): *m/z* calcd for C_25_H_25_N_2_O_10_^+^ (MH)^+^: 513.1504; found: 513.1498.

## Biological evaluations

### Cell culture

Human epidermal keratinocytes (HEKs) were purchased from ThermoFisher Scientific (USA) (cat. no. C0055C) and were grown in EpiLife medium (cat. no. MEPI500CA) supplemented with human keratinocyte growth supplement (HKGS) (cat. no. S0015), according to the manufacturer’s instructions. Cells were passaged at 70–80% confluency, and only cells between passages 1–5 were used in this study. All cell culture reagents were acquired from ThermoFisher Scientific (USA).

### Cytotoxicity assays

The potential cytotoxic effects of novel Nrf-2 activator LN-53 were investigated in both HEKs by MTT, LDH, live/dead cell staining, and caspase-3,-8,-9 multiplex activity assays. Cells were seeded into 96-well plates at a 15,000 cells/well density for all cytotoxicity assays. After cells were allowed to attach overnight, they were treated with increasing concentrations of LN-53 (0.5–25 µM) for 1, 3, 6, and 24 h.

### 3-(4,5-Dimethylthiazol-2-yl)-2,5-diphenyltetrazolium bromide (MTT) assay

MTT (Sigma-Aldrich, Germany) (cat. no. M2128) was used to evaluate cell viability as previously described [29, 30]. Briefly, at the end of the treatment, the medium was discarded, and fresh medium was added to each well. Later, MTT solution (5 mg/ml) was added to the wells, and after a 3-h incubation period, formazan crystals were dissolved in isopropanol. Absorbance values were read by EnSight Multimode Plate Reader (PerkinElmer, USA) at 570 nm.

#### Lactate dehydrogenase (LDH) activity measurement

Cytotoxic effects of LN-53 were assessed by LDH-Cytox™ Assay Kit (cat. no. 426401) (Biolegend, USA) as previously described [29]. Lysis buffer containing Triton X-100 supplied by the kit was used as a positive control. Briefly, at the end of the treatment, the medium in each well was collected and incubated with LDH substrate and the reaction was stopped with the stop solution. LDH content released by the cells was measured by EnSight Multimode Plate Reader (PerkinElmer, USA) at 490 nm.

#### Live/dead cell imaging

Live/dead cell staining was performed further to investigate the effects of LN-53 on cell viability as previously described [29, 30]. Briefly, cells were washed with PBS twice after the treatment and stained with Calcein AM/EthIII solution (cat. no. 30002) (Biotium, USA). After incubation, cells were visualized under an EVoS Floid fluorescent microscope (ThermoFisher, USA).

### Caspase-3, caspase-8, and caspase-9 multiplex activity assay

The potential apoptotic effects of LN-53 were evaluated by caspase-3, -8, and − 9 using the Multiplex Activity Assay Kit (cat. no. ab219915) (Abcam, UK) as previously described [29]. Briefly, cells were treated with increasing concentrations of LN-53, and at the end of the treatment, tri-caspase activity was assayed within the same well by a caspase assay solution containing substrates for caspase-3/7, -8, and − 9 and measured at Ex/Em: 535/620, 490/525, and 370/450 nm, respectively. Cells were also treated with camptothecin (10 µM) for 2 h as a positive control to induce caspase activities, based on our previous work [29].

### Total oxidant status (TOS) measurement

The TOS levels in each sample were measured by TOS Colorimetric Assay Kit (cat. no. E-BC-K802-M) (Elabscience, USA) according to the manufacturer’s instructions. Briefly, various concentrations of hydrogen peroxide (H_2_O_2_) were prepared to construct a standard graph, and the oxidizing capacity within the samples was calculated in terms of µmol H_2_O_2_ equivalent/L [31].

### Total antioxidant status (TAS) measurement

The TAS levels in each sample were measured by TAS Colorimetric Assay Kit (cat. no. E-BC-K801-M) (Elabscience, USA) according to the manufacturer’s instructions. Trolox (as a positive control) was prepared at various concentrations and used as a reference substance for TAS. The TAS levels of the samples were calculated in terms of mmol trolox equivalent/L [31].

### Intracellular ROS measurement

The amount of ROS production in HEKs was measured by using 2′,7′-dichlorofluorescein diacetate (DCFH-DA) (cat. no. D6883) (Sigma-Aldrich, Germany) as previously reported [29]. Briefly, cells were seeded on black 96-well plates (Greiner, Germany) at a 15,000 cells/well density and allowed to attach overnight. Cells were then stained with 10 µM DCFH-DA. After washing with PBS, cells were exposed to increasing concentrations of LN-53 (1–10 µM). Tertbutyl hydroperoxide (TBHP, Luperox^®^ TBH70X, cat. no. 458139) (Sigma-Aldrich, Germany) at 0.05 mM concentration was used as a positive control to induce ROS production. All stimulation conditions were prepared immediately before the stimulation time. The potential effects of LN-53 on TBHP-mediated ROS production were assessed by measuring fluorescence intensity at Ex/Em: 490/525 nm by EnSight Multimode Plate Reader (PerkinElmer, USA).

### SiRNA transient transfection

HEKs (with passage number between 4 and 5) were transfected with Nrf-2 siRNA (cat. no. sc-37030) (Santa Cruz Biotechnology, USA) to suppress Nrf-2 gene expression. HEKs at 70,000 cells/well density were seeded into 24-well plates and allowed to attach overnight. The next day, cells with less than 70% confluency were transfected with Nrf-2 siRNA and Lipofectamine 3000 (cat. no. L3000001) (ThermoFisher Scientific, USA) complex for 24 h in an antibiotic-free medium [32]. Cells were then treated with increasing concentrations of LN-53 (1–5 µM). Lipofectamine 3000 and negative siRNA controls were also included to detect assay-specific alterations.

### Gene expression analysis

The alteration of Nrf-2, HO-1, and NQO1 gene expression profile was investigated as previously described after stimulation with different concentrations of LN-53 [30]. For time-dependent expression profile, HEKs were seeded at 100,000 cells/well density, and the next day, cells were treated with LN-53 agent (1–10 µM) for 1, 3, 6, and 24 h. RNA isolation was performed using PureLink RNA Mini Kit (cat. no. 12183018 A) (ThermoFisher Scientific, USA). cDNA was then synthesized by the RevertAid First Strand cDNA Synthesis Kit (cat. no. K1622) (ThermoFisher Scientific, USA). Gene expression analysis was then performed by PowerUp SYBRGreen Master Mix (cat. no. A25742) (ThermoFisher Scientific, USA) using the Applied Biosystems 7500 Fast device. The primer list used in qPCR is given in Table 1. The PPIA gene was used as an internal reference to normalize the target transcripts using the 2^−ΔΔct^ method.

**Table 1:** Primer sequences of the primer pairs of target genes

Target	Forward primer (3’→5’)	Reverse primer (3’→5’)
Nrf-2	GATATGGTACAACCCTTGTCA	CCAGGACTTACAGGCAATTC
HO-1	GGTCCTTACACTCAGCTTTC	AAGTTCATGGCCCTGGGAG
NQO1	TTCTGTGGGCCATCACTTG	GGAAGCCTGGAAAGATACCCA
PPIA	TCTTTCACTTTGCCAAACACC	CATCCTAAAGCATACGGGTCC

### Protein extraction from nuclear and cytosolic fractions

HEKs were seeded at 500,000 cells/well density in 25 cm^2^ cell culture flasks and treated with LN-53 at 1, 2.5, and 5 µM for 1 and 3 h the next day. After stimulation, nuclear and cytosolic fractions were isolated using a Nuclear Extraction Kit (cat. no. 10009277) (Cayman, USA) according to the manufacturer’s instructions.

### Nrf-2 activation assay

The capability of LN-53 in inducing active Nrf-2 levels in the nucleus was investigated by Nrf2 Transcription Factor Assay Kit (cat. no. 600590) (Cayman, USA) following the manufacturer’s instruction. The total protein content in each sample was quantified by Micro BCA™ Protein Assay Kit (cat. no. 23235) (ThermoFisher, USA). Nrf-2 protein level in each sample was measured at 450 nm by EnSight Multimode Plate Reader (PerkinElmer, USA) [25].

### Western blot analysis

Western blot analysis from cytosolic fractions was performed as previously described [33, 34]. Antibodies against HO-1 (cat. no. sc-136960, 1:500), and NQO1 (cat. no. sc-32793, 1:500) were purchased from Santa Cruz Biotechnology (Texas, USA). β-actin (cat. no. A5441, 1:5,000) (Sigma-Aldrich, USA) antibody was used for the normalization of cytosolic fractions. HRP-conjugated goat anti-mouse IgG secondary antibody (cat. no. 111-035-144, 1:10,000) was obtained from Jackson ImmunoResearch (UK). The membranes were visualized with Clarity Western ECL Substrate (cat. no. 1705061) using ChemiDoc MP Imaging System (Bio-Rad Laboratories, USA).

### Enzyme-linked immunosorbent assay (ELISA)

Cell culture supernatants were collected at the end of the treatment and stored at -80 °C until analysis. Interleukin (IL)-6 and IL-8 cytokine release in the supernatants was quantified by Human IL-6 and Human IL-8 ELISA Kit (cat. no. 88-7066-86 and 88-8086-86, respectively) (ThermoFisher Scientific, USA) as previously described [35].

### Statistical analysis

Statistical analyses for all data were performed using GraphPad Prism 10.0 software (USA). Data were presented as mean ± SEM. One-way ANOVA was performed to compare multiple groups, followed by Dunnett’s post hoc test. Two-way ANOVA was used to analyze multiple groups when independent variables are present, followed by Tukey’s post hoc test. Exact p-values were indicated on each graph. For ROS assay, significant p-values are indicated as follows: *p* < 0.05 (*), *p* < 0.01 (**), *p* < 0.001 (***), and *p* < 0.0001 (****) compared to unstimulated cells and ### *p* < 0.001 and #### *p* < 0.0001 compared to TBHP-stimulated cells.

## Results

### Design, synthesis, and chemical stability of LN-53

**Fig. 1:**
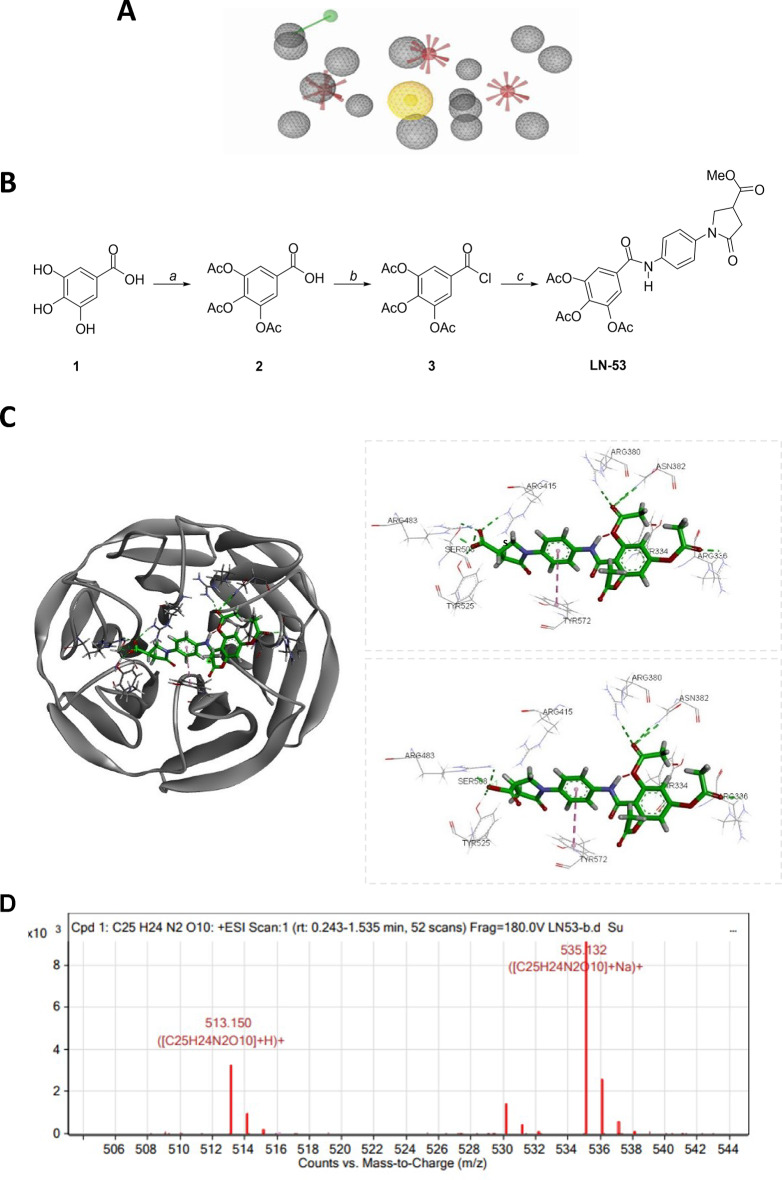
(**A**) The final pharmacophore for the design of the Nrf-2 activators based on non-covalent, electrostatic binding interactions with Keap1. Features are color-coded as follows: negative charge, red; hydrogen bond donor, green; hydrophobic center, yellow; excluded volumes, gray. (**B**) Synthesis of novel amide pyrrolidine derivative LN-53. Reagents and conditions: **a**) gallic acid (1), acetic anhydride, sulfuric acid (cat.), reflux, 4 h. **b**) Thionyl chloride, DMF (cat.), toluene, reflux, 3 h. **c**) methyl 1-(4-aminophenyl)-5-oxopyrrolidine-3-carboxylate, Et_3_N, DMAP (cat.), dichloromethane, r.t., 12 h. (**C**) Docking pose of R and S enantiomers of LN-53 (green), its amino acid interactions with the binding site of Keap1, and their 2D ligand interaction diagrams. Hydrogen bonds are indicated by dashed lines. (**D**) Stability assessment of LN-53: LC-MS results of LN-53. 10 mg of LN-53 was initially dissolved in a minimal volume of DMSO (1 ml), with subsequent addition to water at pH 5.5. The resulting solution was incubated at 37 °C for 24 h prior to LC-MS analysis

The pharmacophore for non-covalent electrostatic binding to Keap1 (Fig. 1A) was used to design a series of compounds, including the parent lead compound SK-119 (Scheme S1) was reported previously [36]. LN-53 was designed and synthesized as a racemate using the pharmacophore model (Fig. 1B). The original synthesis of SK-119 consisted of mono-cyclization of *p*-phenylenediamine (S-2) with itaconic acid (S-1) to give pyrrolidine-3-carboxylic acid S-3 (85%), which subsequently reacted with 2,4,6-trihydroxybenzaldehyde (S-4) with formation of SK-119 (39%) (Scheme S1) [36]. Thus, the original route to SK-119 required the use of excess highly carcinogenic S-2 and laborious isolation of the intermediate (S-3) by preparative HPLC.

The synthesis of LN-53 started from commercially available inexpensive 3,4,5-trihydroxybenzoic acid (gallic acid) (1) (Fig. 1B). Alike the polyhydroxylated fragment in the parent lead SK-119 gallic acid (1) possesses three phenolic hydroxyles in one aryl ring. However, in 1 the phenolic functionalities are arranged contiguously in *metha*- and *para*- positions 3, 4, and 5 of the benzene ring, dissimilar to the arrangement of phenolic hydroxyles in SK-119 presented in *ortho*- and *para*-positions 2, 4, 6- of the benzene ring. Nevertheless, due to the similarity of the functional groups and the overall structural features capable in the former pharmacological profile, the gallic acid (**1**) fragment was selected as a reasonable structural pharmacophore. All three phenols in gallic acid (**1**) were protected with acetyl groups according to the reported sulfuric acid-catalyzed acetylation with acetic anhydride upon heating to afford solid triacetoxy-derivative **2** [37–40]. Subsequent treatment of **2** with excess thionyl chloride in the presence of DMF as a catalyst led to 3,4,5-triacetoxybenzoyl chloride (**3**), which was obtained in a crude state after removal of all volatile components from the reaction mixture under vacuum [37, 38, 41]. Subsequent reaction between methyl 1-(4-aminophenyl)-5-oxopyrrolidine-3-carboxylate and crude acyl chloride **3** in the presence of triethylamine and DMAP (cat.) resulted in the stable amide LN-53 (Fig. 1B), a stabilized analogue of SK-119 (Scheme S1). Analytical structural data of LN-53 are presented in Figure S1.

The docking of two LN-53 enantiomers to the 3D Nrf-2/Keap1 model was conducted for the determination of the more suitable isomer for binding to Keap1 (Fig. 1C). Compound LN-53 revealed in silico interactions with the amino acids of the Keap1 binding site with a relatively higher docking score and forms H-bond interactions with residues ARG336, ASN380, ASN382, ARG415, ARG483, SER508, TYR525, and π-π stacking interactions with TYR525. In addition, hydrophobic interactions with PHE478, GLY509, GLN530, ALA556, PHE577, SER602 were determined with docking score − 8.78 (Fig. 1C). The parent compound SK-119 forms fewer H-bond interactions with residues of Keap1 [25]. Interestingly, both enantiomers exhibited similar binding poses and interacted with the same key residues. The only observed difference was the absence of a single hydrogen bond interaction with residue ARG380 in the *R*-enantiomer of LN-53. Despite this variation, the binding energies of both enantiomers were comparable. Interestingly, the docking scores, which estimated the binding affinity between molecules, for LN-53 were significantly higher than those of SK-119.

LN-53 successfully underwent chemical stability testing to simulate the pH conditions in the skin, being exposed to pH 5.5 and a temperature of 37 °C for 24 h. Following this exposure period, the possible disintegration of the molecule was tested by using thin-layer chromatography (TLC) and liquid chromatography-mass spectrometry (LC-MS) methods (Fig. 1D). LN-53 exhibited exceptional stability during the entire experimental duration, as evidenced by the absence of decomposition observed in the TLC and LC-MS results. Indeed, in Fig. 1D shows that only a protonated molecular peak (M + H)^+^ and the corresponding peak (M + Na)^+^ of LN-53 are present in the LC-MS analysis, whilst no other ions (the signs of the degradation) have appeared. Also, in TLC analysis, no additional UV-active spots were observed. In contrast to that, the identical experimental conditions led to a complete decomposition of the parent compound SK-119 after 30 min.

### LN-53 did not exert significant cytotoxic effects

**Fig. 2:**
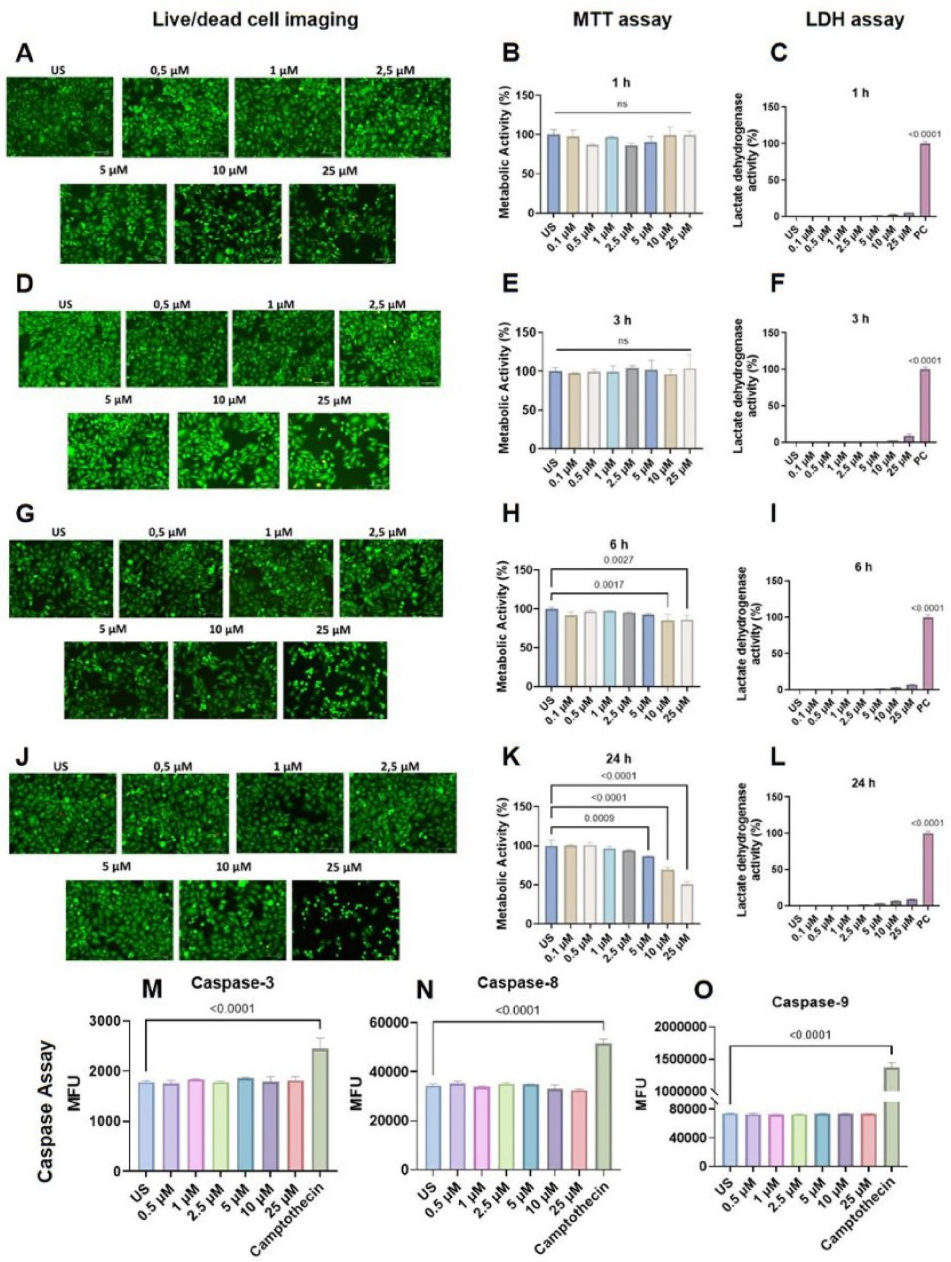
Cytotoxic effects of LN-53 on human epidermal keratinocytes (HEKs). Live/dead cell staining (**A**, **D**, **G**, **J**), MTT analyses (**B**, **E**, **H**, **K**), LDH release levels (**C**, **F**, **I**, **L**), and multiplex caspase activity analyses (**M**, **N**, **O**) were employed to assess potential cytotoxicity of LN-53. Lysis buffer containing Triton X-100 was used as a positive control for the LDH assay. Camptothecin (10 µM) was used as a positive control for the multiplex caspase assay to induce caspase activities. Data were analyzed by ordinary one-way ANOVA followed by Dunnett’s multiple comparisons test. Exact p-values were indicated on graphs. Bars represent means, while whiskers represent standard error of the mean (SEM). Assays were performed in triplicate (*n* = 3). US: unstimulated, PC: positive control (lysis buffer), ns: non-significant

HEK cells treated with LN-53 at concentrations ranging from 0.5 to 25 µM did not demonstrate significant cytotoxicity, except 25 µM concentration (Fig. 2). Live/dead cell imaging showed a morphological change in HEKs at the highest concentration (25 µM) after 6 h, and this observation was confirmed with MTT analysis, which indicated a decrease after 10 µM concentration (Fig. 2G and H, ordinary one-way ANOVA, F_7, 16_=5.584, *p* = 0.0021). According to MTT analysis, a significant decline in metabolic activity was observed starting from 5 µM LN-53 treatment after 24-hour treatment (Fig. 2K, ordinary one-way ANOVA, F_7, 16_=87.11, *p* < 0.0001). However, live/dead cell staining showed cytotoxicity only at the highest concentration, 25 µM. LDH assay and multiplex caspase measurement showed no significant cell death in HEKs (Fig. 2C, F and I, and 2L, ordinary one-way ANOVA, F_8, 18_=3247, *p* < 0.0001, F_8, 18_=2406, *p* < 0.0001, F_8, 18_=3235, *p* < 0.0001, and F_8, 18_=3214, *p* < 0.0001, respectively). The 25 µM concentration was excluded from further experiments. All statistics information for Fig. 2, including F_DFn, DFd_ and p-values of the test, is presented in the Supplementary file.

### LN-53 effectively suppressed TBHP-mediated intracellular ROS production

**Fig. 3:**
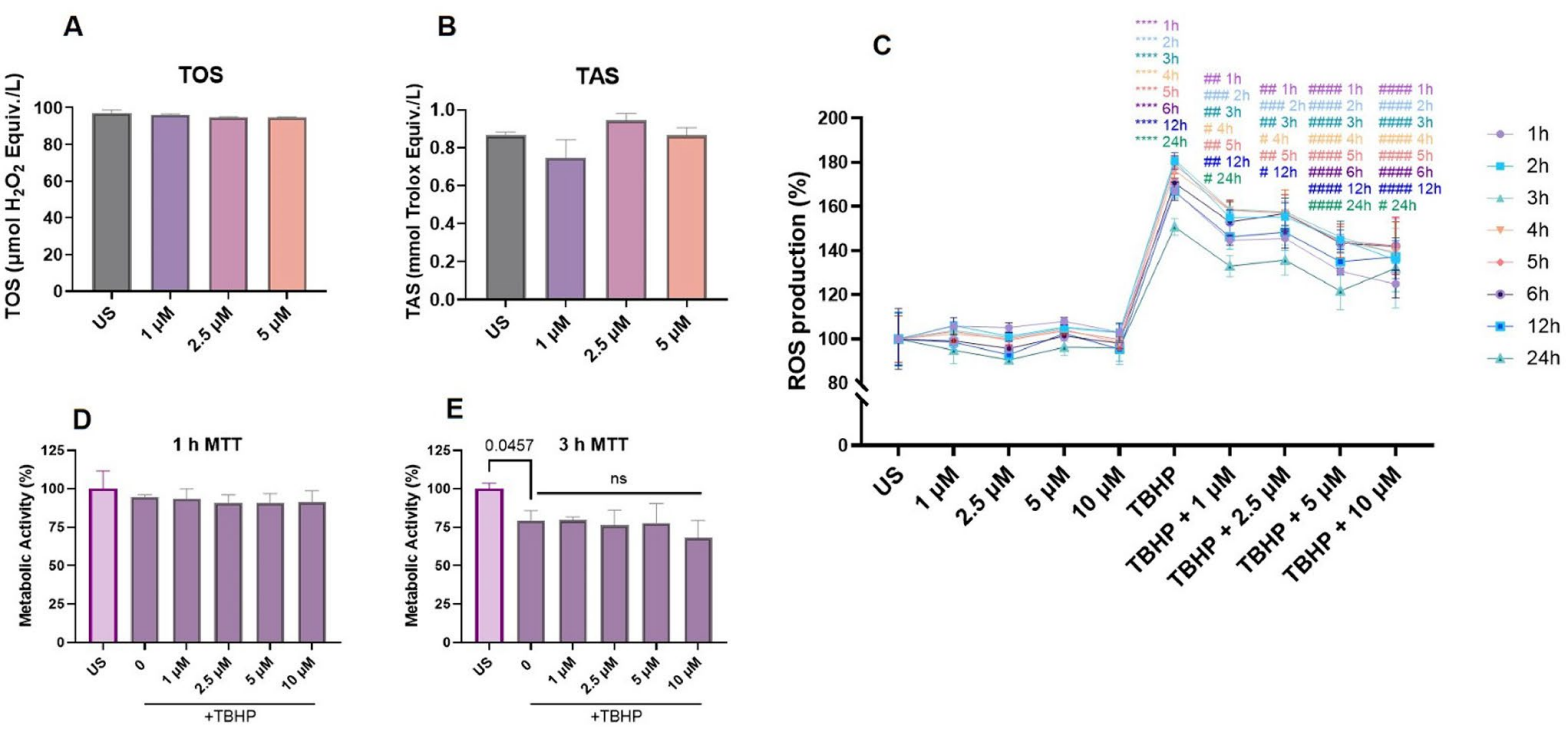
The effect of LN-53 on total oxidant status (TOS) (**A**), total antioxidant status (TAS) (**B**), tBHP-mediated and intracellular ROS production (**C**), and cell viability during TBHP-stimulation (**D**;1-hour and **E**;3-hour) in HEKs. For TOS, TAS, and MTT assays, data were analyzed by ordinary one-way ANOVA followed by Dunnett’s multiple comparisons test. Exact p-values were indicated on graphs. Bars represent means, while whiskers represent standard error of the mean (SEM). For the reactive oxygen species (ROS) production assay, data were analyzed by ordinary two-way ANOVA followed by Tukey’s multiple comparisons test. Symbols represent means, while whiskers represent standard error of the mean (SEM). Assays were performed in triplicate (*n* = 3). US: unstimulated cells, tBHP: tertbutyl hydroperoxide. **** *p* < 0.0001 compared to unstimulated cells, # *p* < 0.05, ## *p* < 0.01, ### *p* < 0.001 and #### *p* < 0.0001 compared to TBHP-stimulated cells

LN-53 treatment alone did not significantly alter the total oxidant status (TAS) or the total antioxidant status (TOS) in HEKs (Figs. 3A, ordinary one-way ANOVA, F_3_, _8_=3.350, *p* < 0.0762 and Fig. 3B, ordinary one-way ANOVA, F_3, 8_=6.299, *p* = 0.0168). Consistent with this finding, LN-53 stimulation alone did not affect ROS production at any concentration and treatment duration (Fig. 3C, ordinary two-way ANOVA effect of stimulation F_9, 60_=404.5, *p* < 0.0001) and time F_7, 160_=15.61, *p* < 0.0001), with interaction F_63, 160_=1.344, *p* = 0.0718)). TBHP, on the other hand, the increased intracellular ROS level, even beginning from one hour of stimulation. The introduction of LN-53 into the environment reduced the TBHP-induced ROS production in a concentration-dependent fashion. We also analyzed the potential effect of LN-53 on the cell viability of TBHP-stimulated cells. After 1-hour treatment, no difference was observed in cell viability (Fig. 3D, ordinary one-way ANOVA, F_5, 12_=0.7556, *p* = 0.5982), whereas TBHP decreased cell viability to nearly 80% (Fig. 3E, ordinary one-way ANOVA, F_5, 12_=4.603, *p* = 0.0141). Although LN-53 showed a significant effect on protecting cells against TBHP-mediated ROS production, it did not exert any protective effect on cell viability.

### The expression levels of Nrf-2 and related genes were induced upon LN-53 treatment

**Fig. 4:**
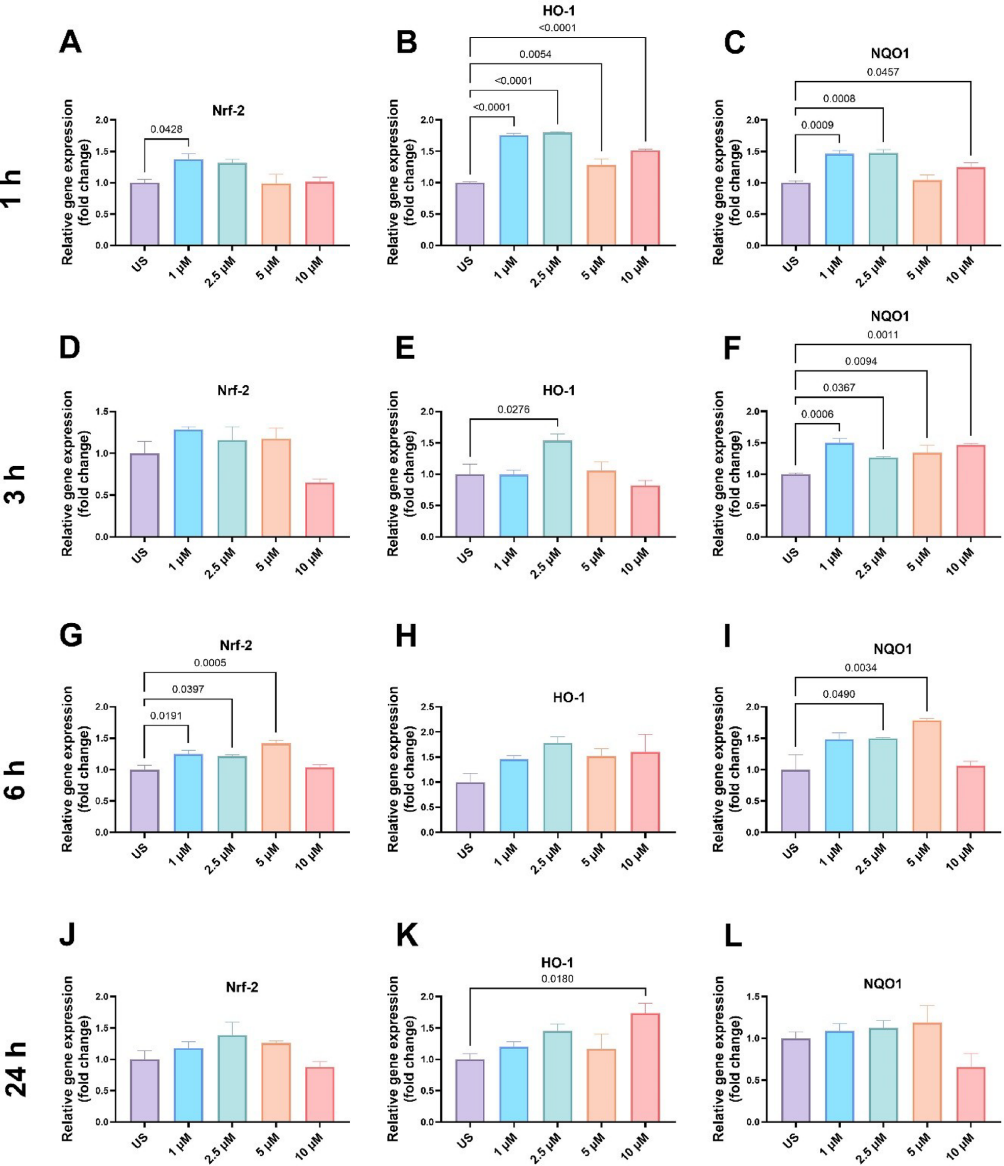
The gene expression levels of Nrf-2 related genes in response to LN-53 treatment in HEKs after 1, 3, 6, and 24 h. Gene expression levels were measured by quantitative polymerase chain reaction (qPCR). Data were analyzed by ordinary one-way ANOVA followed by Dunnett’s multiple comparisons test. Exact p-values were indicated on graphs. Bars represent means, while whiskers represent standard error of the mean (SEM). Assays were performed in triplicate. Lipofectamine 3000 reagent and negative control siRNA were used to assess potential confounding effects. US: Unstimulated

The 1-hour treatment with LN-53 caused a slight increase in Nrf-2 gene expression at 1 µM concentration (Fig. 4A, ordinary one-way ANOVA, F_4, 10_=4.542, *p* = 0.0238), while the HO-1 transcription level was induced at all concentrations (Fig. 4B, ordinary one-way ANOVA, F_4, 10_=50.90, *p* < 0.0001). NQO1 gene expression was also increased at all concentrations except 5 µM (Fig. 4C, ordinary one-way ANOVA, F_4_, _10_=14.07, *p* = 0.0004). Although 3-hour LN-53 exposure did not affect Nrf-2 gene expression in HEKs (Fig. 4D, ordinary one-way ANOVA, F_4, 10_=4.765, *p* = 0.0207) and only a slight increase was observed in HO-1 level at 2.5 µM concentration (Fig. 4E, ordinary one-way ANOVA, F_4, 10_=5.329, *p* = 0.0146), NQO1 transcription level was induced after all concentrations (Fig. 4F, ordinary one-way ANOVA, F_4, 10_=10.49, *p* = 0.0013). Nrf-2 and NQO1 gene expression increasing patterns were similar after 6-hour LN-53 stimulation (Fig. 4G, ordinary one-way ANOVA, F_4, 10_=11.50, *p* = 0.0009 and Fig. 4I, ordinary one-way ANOVA, F_4, 10_=7.372, *p* = 0.0049). Although 6-hour treatment with LN-53 agent caused around 1.5X fold increasing trend in HEK cells, no statistical significance was observed in HO-1 gene expression (Fig. 4H, ordinary one-way ANOVA, F_4, 10_=2.218, *p* = 0.14). Unlike early stimulations, 24-hour treatment with LN-53 did not affect Nrf-2 and NQO1 gene expression levels in HEKs (Fig. 4J, ordinary one-way ANOVA, F_4, 10_=2.584. *p* = 0.1018 and Fig. 4L, ordinary one-way ANOVA, F_4, 10_=2.508, *p* = 0.1087), while only 10 µM concentration induced HO-1 transcription level (Fig. 4K, ordinary one-way ANOVA, F_4, 10_=3.775, *p* = 0.0402).

### Nrf-2 gene silencing blocked LN-53-mediated alterations in gene expression

**Fig. 5:**
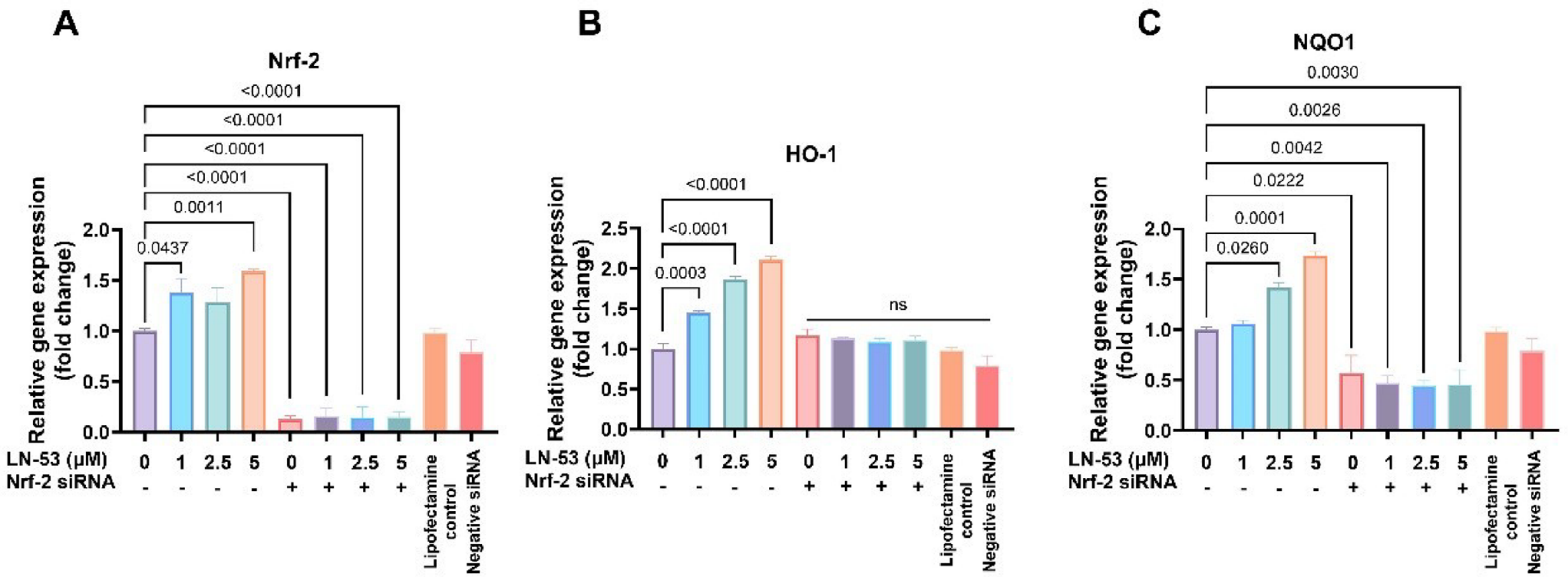
The gene expression levels of Nrf-2-related genes in response to 6-hour LN-53 treatment in Nrf-2-knockdown HEKs. Gene expression levels were measured by quantitative polymerase chain reaction (qPCR). Data were analyzed by ordinary one-way ANOVA followed by Dunnett’s multiple comparisons test. Exact p-values were indicated on graphs. Bars represent means, while whiskers represent standard error of the mean (SEM). Lipofectamine 3000 reagent and negative control siRNA were used to assess potential confounding effects. Assays were performed in triplicate (*n* = 3). US: Unstimulated

The knockdown of Nrf-2 through siRNA transfection led to a reduction of over 90% in Nrf-2 transcription levels in HEKs (Fig. 5A, ordinary one-way ANOVA, F_9, 20_=41.48, *p* < 0.0001). Although LN-53 induced Nrf-2 gene expression on its own, it could not compensate for the effect of gene silencing, which drastically suppressed Nrf-2 mRNA levels. We did not observe any significant change in transfection reagent and negative siRNA controls compared to unstimulated cells. Although upto 1.7-fold increase observed in HO-1 expression after 6-hour treatment with LN-53, which was not statistically significant (Fig. 4H), we observed a dose-dependent increase in HO-1 gene expression upon LN-53 stimulation was blocked by Nrf-2 gene silencing (Fig. 5B, ordinary one-way ANOVA, F_9, 20_=46.64, *p* < 0.0001). Nrf-2 siRNA transfection on its own suppressed NQO1 transcription by almost 40% (Fig. 5C, ordinary one-way ANOVA, F_9, 20_=22.95, *p* < 0.0001). The increases observed in NQO1 gene expression after 2.5 and 5 µM LN-53 stimulation were completely blocked by Nrf-2 knockdown, and a very similar response was observed in Nrf-2 gene expression.

### Nuclear translocation of Nrf-2 was induced by LN-53 treatment

**Fig. 6:**
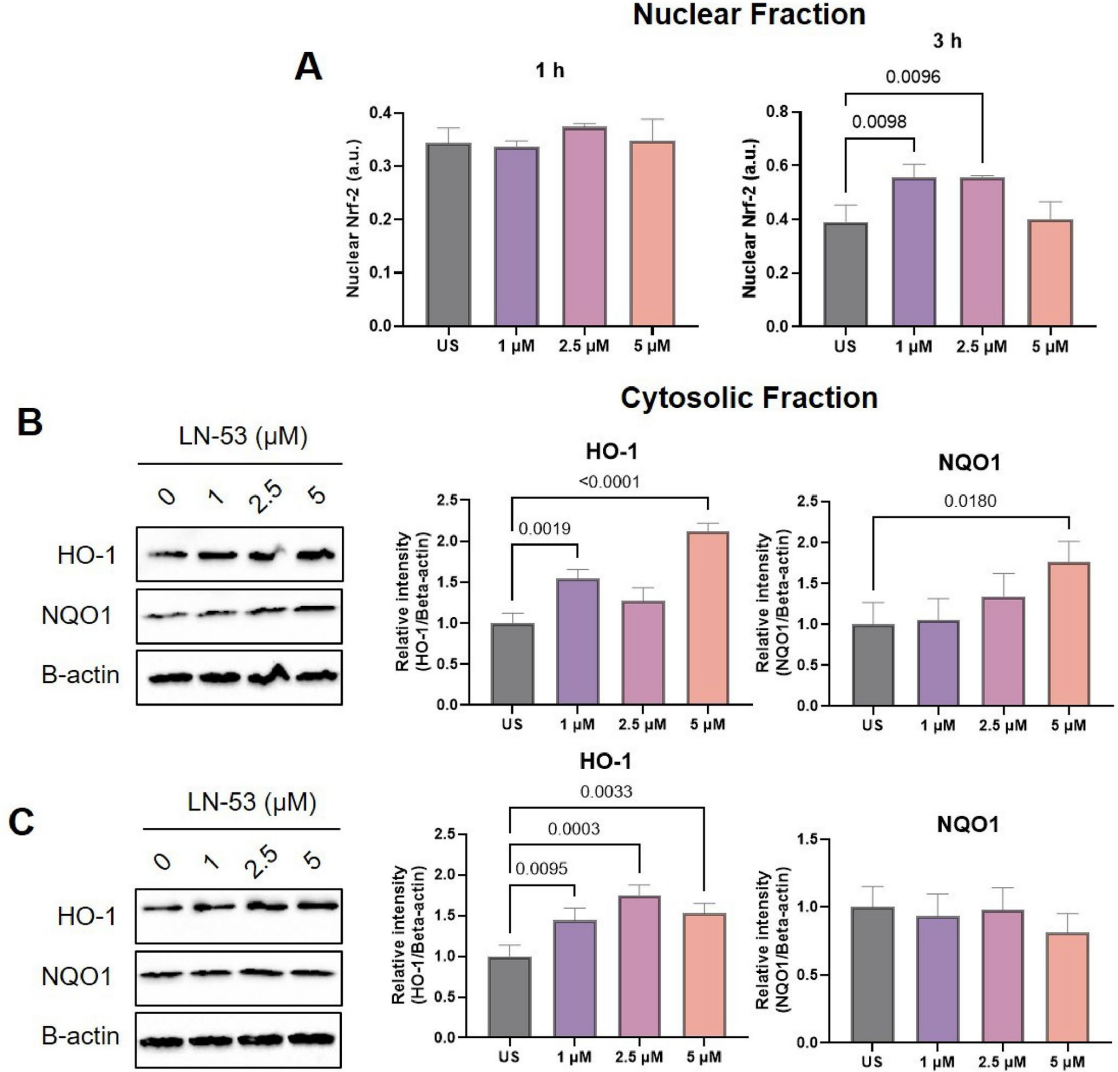
The effect of LN-53 treatment on the activation of the Nrf-2 pathway. After stimulations, nuclear and cytosolic fractions were isolated from the cells. Nrf-2 level in nuclear fractions of HEKs after 1 and 3 h was determined by Nrf-2 activity assay (**A**). HO-1 and NQO1 protein levels in cytosolic fractions in HEKs by Western blot analysis after 1-hour (**B**) and 3-hour (**C**) stimulation. Band intensities were measured by ImageJ software. Data were analyzed by ordinary one-way ANOVA followed by Dunnett’s multiple comparisons test. Exact p-values were indicated on graphs. Bars represent means, while whiskers represent standard error of the mean (SEM). All biological assays were performed in triplicate (*n* = 3). US: Unstimulated. a.u.: absorbance unit

Nrf-2 translocation was assessed by determining the Nrf-2 protein level in the nuclear fractions isolated from treated cells using an ELISA-based Nrf-2 assay. We found that 1-hour treatment with LN-53 did not alter the Nrf-2 level in the nucleus (ordinary one-way ANOVA, F_3, 8_=1.237, *p* = 0.3584), while active Nrf-2 level found in nuclear fractions were induced at 1 and 2.5 µM concentrations in after 3-hour treatment, although 5 µM concentration did not establish a significant change (Fig. 6A, ordinary one-way ANOVA, F_3, 8_=10.10, *p* = 0.0043).

Nrf-2 activation level was also assessed by the analysis HO-1, and NQO1 protein levels in the cytosolic fractions using Western blot. We observed that Nrf-2 protein levels in cytosolic fractions were induced after 2.5 and 5 µM concentrations after 1-hour treatment with LN-53 (Fig. 6B, ordinary one-way ANOVA, F_3, 8_=188.8, *p* < 0.0001). HO-1 protein level was induced after 1 and 5 µM concentrations (ordinary one-way ANOVA, F_3,8_=1.237, *p* < 0.0001), while only an increase in NQO1 protein level was detected at 10 µM concentration after 1-hour stimulation (Fig. 6B, ordinary one-way ANOVA, F_3,8_=5.375, *p* = 0.0255). Unlike 1-hour exposure, we observed no change in Nrf-2 (ordinary one-way ANOVA, F_3, 8_=3.958, *p* = 0.0531) and NQO1 (ordinary one-way ANOVA, F_3, 8_=0.8921, *p* = 0.4858) protein levels in cytosolic fractions after 3-hour treatment with LN-53, although the HO-1 protein level was induced at all concentrations (ordinary one-way ANOVA, F_3, 8_=16.75, *p* = 0.0008) (Fig. 6C).

### LN-53 treatment suppressed IL-6 and IL-8 cytokine release

**Fig. 7:**
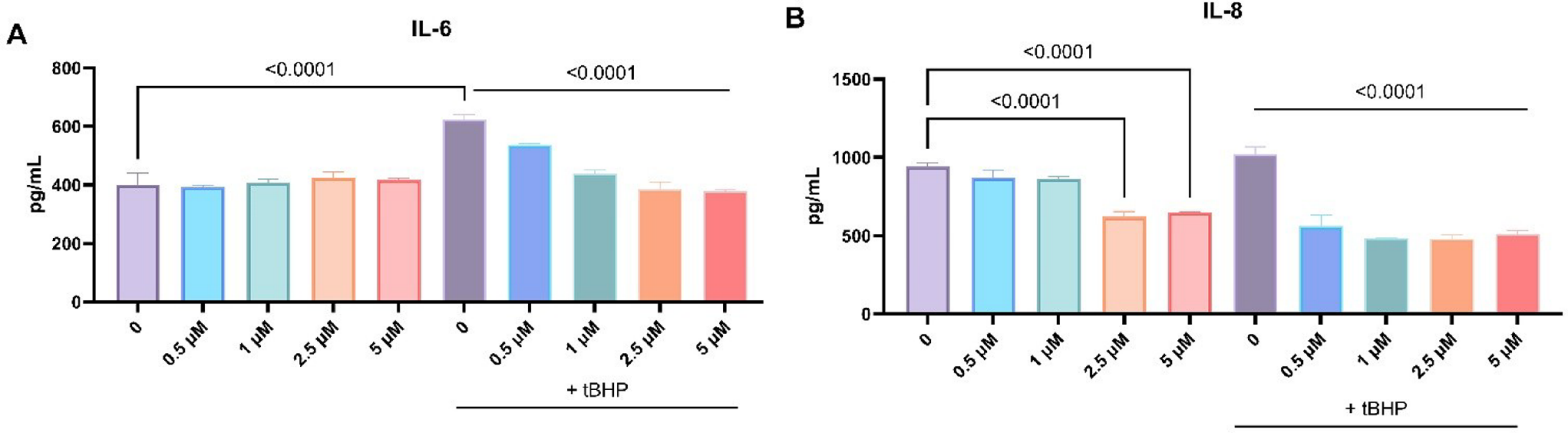
The effect of LN-53 treatment on IL-6 (**A**) and IL-8 (**B**) cytokine production in HEKs after 6 h. After 6 h-treatment, cytokine production in the cell culture supernatants was quantified by ELISA. Data were analyzed by ordinary one-way ANOVA followed by Dunnett’s multiple comparisons test. Exact p-values were indicated on graphs. Bars represent means, while whiskers represent standard error of the mean (SEM). Assays were performed in triplicate (*n* = 3)

6-hour treatment with LN-53 alone did not affect IL-6 cytokine production, while TBHP treatment significantly induced IL-6 release. tBHP-mediated increase in IL-6 production was suppressed by LN-53 stimulation in a dose-dependent manner (Fig. 7A, ordinary one-way ANOVA, F_9, 20_=57.01, *p* < 0.0001). IL-8 cytokine production was lowered by 2.5 and 5 µM concentrations of LN-53. Although TBHP did not affect IL-8 release in HEKs on its own, LN-53 exposure significantly reduced IL-8 production compared toTBHP-stimulated cells at all concentrations (Fig. 7B, ordinary one-way ANOVA, F_9, 20_=99.56, *p* < 0.0001).

## Discussion

Nrf-2-KEAP1 sensor-effector machinery is a major player in maintaining redox balance, especially under stress conditions [42, 43]. This signaling pathway is crucial to protect cells and tissues from oxidative damage caused by a wide variety of factors, including environmental irritants, especially in the skin, as it induces the production of frontline antioxidant proteins and enzymes. Due to the various beneficial effects of Nrf-2, it has gained special attention as a promising therapeutic target for various oxidative stress-related disorders, including skin diseases. In this study, we evaluated the potential in vitro safety and efficacy of a novel Nrf-2 activator, LN-53, a gallic acid (1) derivative, which could be considered as a structurally modified counterpart of our previously published lead SK-119, on human epidermal keratinocytes. We showed that LN-53 can efficiently increase Nrf-2 intranuclear levels and augment the activity and the levels of molecules related to the Nrf-2 pathway without causing significant cellular damage.

In view of the chemical instability of the parent compound SK-119, which suffers from the imine bond hydrolysis to the corresponding amine (aniline) and aldehydes [44, 45], we decided to replace the imine bond in SK-119 with a more stable amide bond under mild acidic conditions. This structural change left the pharmacophore in the original place but allows to obtain a more stable compound. To this end, we focused the synthesis on increasing the number of rings while avoiding linear derivatives since our earlier research demonstrated that incorporation of cyclic motifs enhances rigidity, which in turn boosts the molecular affinity for Keap1 and improves the pharmacokinetic properties of compounds [10]. Therefore, we incorporated two aromatic and one aliphatic rings in the structure of LN-53. Docking the two possible LN-53 enantiomers to the 3D Nrf-2/Keap1 in silico model showed that both enantiomers exhibited similar binding poses and interacted with the same key residues. Most importantly, the binding energies of both isomers were almost identical. This similarity could be attributed to the use of rigid molecular structures for the docking simulations, which limit protein flexibility and might not capture subtle differences. More advanced techniques like long molecular dynamic simulations could provide a more detailed picture, but such methods were beyond the scope of the present study. Overall, insilico analysis suggests that both SK-119 and LN-53 have the potential to bind to Keap1. Interestingly, the docking scores, which estimate the binding affinity between molecules, for LN-53 were significantly higher than those of SK-119. This enhanced affinity for Keap1 of LN-53 might be attributed to a greater number of hydrogen bonds formed between the compound and the protein. These in silico findings are remarkably consistent with biological experimental data, which demonstrated that LN-53 exhibits superior activity compared to its parent compound SK-119.

Gallic acid (**1**), also known as 3,4,5-trihydroxybenzoic acid, the key scaffold of our compound, is one of the most important polyphenols widely distributed in grapes, mango, and green tea. Like other polyphenols, gallic acid exerts antioxidant and anti-inflammatory activities, regulates redox metabolism and inflammation, and involves various biological processes, including hypertension, tissue remodeling, and fibrosis [46, 47]. Hu et al. showed that gallic acid had a potential therapeutic effect on the skin of mice with atopic dermatitis, reducing dermatitis score and inflammation [48]. Researchers showed that gallic acid (1) did not reduce cell viability up to 200 µM, while higher concentrations induced cell death in both the human epidermal keratinocyte cell line, HaCat, and human dermal fibroblasts (HDFs) [49]. Another study found that gallic acid had no effect on cell viability in HaCaT cells up to 30 µM, whereas cell death started at higher concentrations [50]. Conversely, Zhang et al. recently revealed that gallic acid (1) can initiate apoptosis and decrease cell proliferation starting from 10 µM concentration in IL-17 A-treated HaCaT cells [51]. We showed that LN-53 did not induce significant cell death in HEKs until 24-h treatment at a 25 µM concentration.

Gallic acid (1) suppressed the ROS level induced by tumor necrosis factor (TNF)-α and interferon (IFN)-γ, but this effect was highly limited. While TNF-α and IFN-γ increased ROS production by 300%, the ROS level detected in 30 µM gallic acid-treated HaCaT cells was nearly 280% of the control [50]. Chen et al., on the other hand, showed that gallic acid significantly induces intracellular ROS levels in mouse lung fibroblasts, but they used a higher concentration of gallic acid (nearly 300 µM) than the rest of the literature [52]. Our data showed that intracellular ROS production induced by TBHP, a potent activator of oxidative stress, was significantly suppressed by LN-53 in a concentration and time-dependent manner in human epidermal keratinocytes. We suppose that acetylated gallic acid carboxylic groups might undergo hydrolysis and contribute to the antioxidative effect of LN-53. However, although our findings demonstrate that LN-53 effectively suppresses TBHP-mediated ROS production, indicating its potential as an antioxidant, it did not reverse the detrimental effects of TBHP on cell viability, which may suggest that the antioxidant capacity of LN-53 may be insufficient to restore cell viability.

Resveratrol, another natural polyphenol derived from a diverse variety of fruits and nuts, has been shown to exert its antioxidant activity through Nrf-2 activation [53]. A study showed that resveratrol increased nuclear Nrf-2 levels after 9- and 12-hour treatment at 50 µM concentration in a hepatocellular carcinoma cell line, HepG2, while it was unable to change Nrf-2 amount in the nuclear fractions after 6 h [54]. Another study used 8 µM resveratrol as an Nrf-2 activator and found a significant increase in Nrf-2 translocation in Pancreatic β-cells (MIN6) after 24-hour exposure [55]. Kode et al. also showed that nuclear Nrf-2 level was induced by 10 µM resveratrol after 24 h in A549 cells, however, resveratrol was unable to recover the physiological level when cells were silenced with Nrf-2 siRNA [56]. Sulforaphane (SFN), recognized as a natural activator of Nrf-2, has demonstrated the ability to increase nuclear Nrf-2 levels twofold in the HUVEC cell line when administered at a concentration of 1 µM. The study also conducted in vivo experiments showing that the effect of SFN on aorta endothelial cells depended on the tissue region. While SFN effectively induced nuclear Nrf-2 in the susceptible site of the aorta, while it did not exert a significant change in the protected site [57]. Another study showed that SFN did not change nuclear or cytoplasmic Nrf-2 levels in the hippocampus on its own, but it can only induce Nrf-2 translocation when SFN treatment was combined with chronic intermittent hypoxia [58]. Kubo et al. showed that 6-hour SFN treatment induces nuclear Nrf-2 level in human lens epithelial cells, but not cytosolic Nrf-2, in a concentration-dependent manner, starting from 1 µM [59]. Moghadam et al. performed a study assessing the effects of resveratrol, gallic acid, and kuromanin chloride on Nrf-2/SIRT pathway at 10, 20, and 40 µM concentrations in HepG2 cells after 72-hour treatment [60]. They showed that although resveratrol and kuromanin chloride induced Nrf-2 gene expression in a concentration-dependent manner, gallic acid showed the highest increase at 10 µM, and over this concentration, the Nrf-2 transcriptional level started to drop. The pattern of protein levels of antioxidant enzymes (GSH and SOD) in gallic acid-treated cells was similar to the Nrf-2 gene expression profile. In our study, LN-53 started to induce Nrf-2 activity and the expression levels of Nrf-2 and Nrf-2-related genes (HO-1 and NQO1) at lower concentrations after 6-hour stimulation in HEKs. We revealed that this effect of LN-53 occurred specifically through the interaction with Nrf-2/KEAP1 complex, by showing that LN-53 exerts such an effect only in cells in which Nrf-2 is present but not in Nrf-2-knockdown cells.

Besides the pivotal role in redox regulation, Nrf-2 has also been shown to regulate inflammatory processes. Nrf-2 deficiency can induce aggressive inflammation through NF-κB activation, triggering the production of proinflammatory cytokines [61, 62]. Hu et al. showed that gallic acid treatment in mice with atopic dermatitis decreased the gene expression level of inflammatory cytokines, such as IL-4, TNF-α, and IL-17 [48]. In our previous report, the parent compound SK-119 effectively suppressed IL-8 inflammatory cytokine-induced by diesel particulate matter (DPM) [25]. In the current study, we also observed that LN-53 could diminish the release of both IL-6 and IL-8 cytokines under both normal and TBHP-stimulated environments.

There are several limitations to this study. The major limitation of this study is that the cell culture experiment results were not verified in an animal model. Although the cell culture experiments elucidate the general mechanistic route, it potentially cannot be observed evidently in vivo. Further experiments should be performed with human-skin equivalents and mouse models, which may reflect the physiological processes better than monoculture experiments. Therefore, the observations presented in this paper should be extrapolated carefully. Secondly, our findings showed that the LN-53 agent could exert antioxidant activities in a relatively narrow range in terms of concentrations, as we observed cytotoxicity at higher concentrations. Another limitation in our study was that Nrf2 activation was examined by assessing ROS production, Nrf-2 nuclear translocation, and the expression profiles of Nrf-2 and its associated molecules, HO-1 and NQO1, at both the gene and protein levels. Transcriptomic or proteomic studies may clarify whether the effects of LN-53 are valid for all redox or antioxidant metabolism, or the observed effects are strictly dependent on Nrf-2/KEAP1 pathway and direct interaction between LN-53 and Keap1.

Overall, our results suggest that our newly synthesized Nrf-2 activator LN-53 can efficiently reduce oxidative stress through the Nrf-2 signaling pathway without damaging the cells. The antioxidant activity of LN-53 was strictly dependent on the Nrf-2 presence in the cells, as shown in the Nrf-2 knockdown assays, suggesting that LN-53 may regulate the redox metabolism by acting as a Nrf-2-specific activator. Besides the protective effect on oxidative stress, LN-53 also reduces TBHP-mediated inflammatory response. However, the determination of the direct interaction with Keap1 and further in vivo studies should be performed to make a step forward in the development of a novel pharmacologically relevant antioxidative compound.

## Electronic supplementary material

Below is the link to the electronic supplementary material.

Supplementary Material 1

## Data Availability

Data is provided within the manuscript or supplementary information files can be obtained from authors upon request.
