# Supplementary material for: A novel 2-oxopyrrolidine derivative (LN-53) efficiently induces Nrf-2 signaling pathway activation in human epidermal keratinocytes
Source: Pharmacol Rep. 2025 Jun 24;77(5):1347–63. doi: 10.1007/s43440-025-00757-y (PMC12443944; doi:10.1007/s43440-025-00757-y)
Supplement: Supplementary file 1 — Supplementary Material 1 [file 43440_2025_757_MOESM1_ESM.docx]

**Supplementary File**

**A novel 2-oxopyrrolidine derivative (LN-53) efficiently induces Nrf-2 signaling pathway activation in human epidermal keratinocytes**

Basak Ezgi Sarac^1^*, Laura Nissim^2^*, Dilara Karaguzel^1^, Gokhan Arik^1^, Shirin Kahremany^2^, Edward E. Korshin^2^, Arie Gruzman^2**^, Cagatay Karaaslan^1**^

* Basak Ezgi Sarac and Laura Nissim should be considered joint first authors.

** Arie Gruzman and Cagatay Karaaslan should be considered joint senior authors.

^1^ Department of Biology, Molecular Biology Section, Faculty of Science, Hacettepe University, Ankara, Turkey

^2^ Department of Chemistry, Faculty of Exact Sciences, Bar-Ilan University, Ramat-Gan, Israel

**Scheme S1.** Synthesis of SK-119. Reagents and conditions: a) (i) H_2_O, reflux, 1 h; (ii) HPLC purification. b) EtOH, r.t., overnight.

**Analytical structural data of LN-53: C_25_H_24_N_2_O_10_ (Brutto-formula C_25_H_24_N_2_O_10_)**


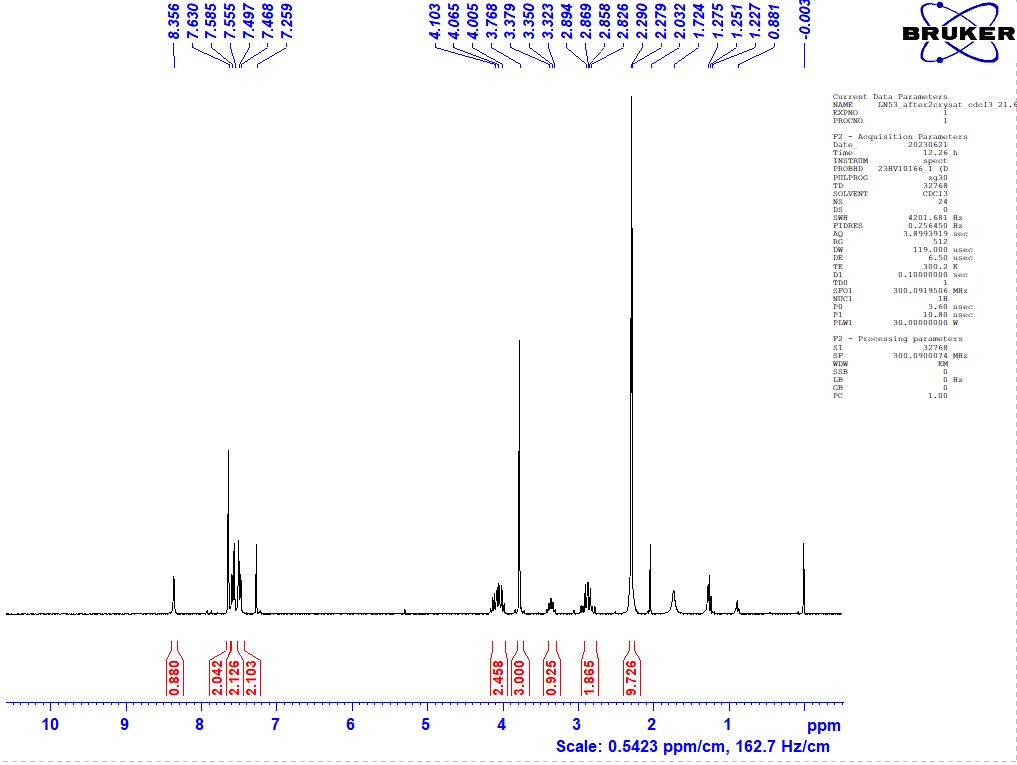
 ¹H NMR

**Figure S1a.** ¹H NMR spectrum of LN-53 (CDCl₃, 300 MHz).


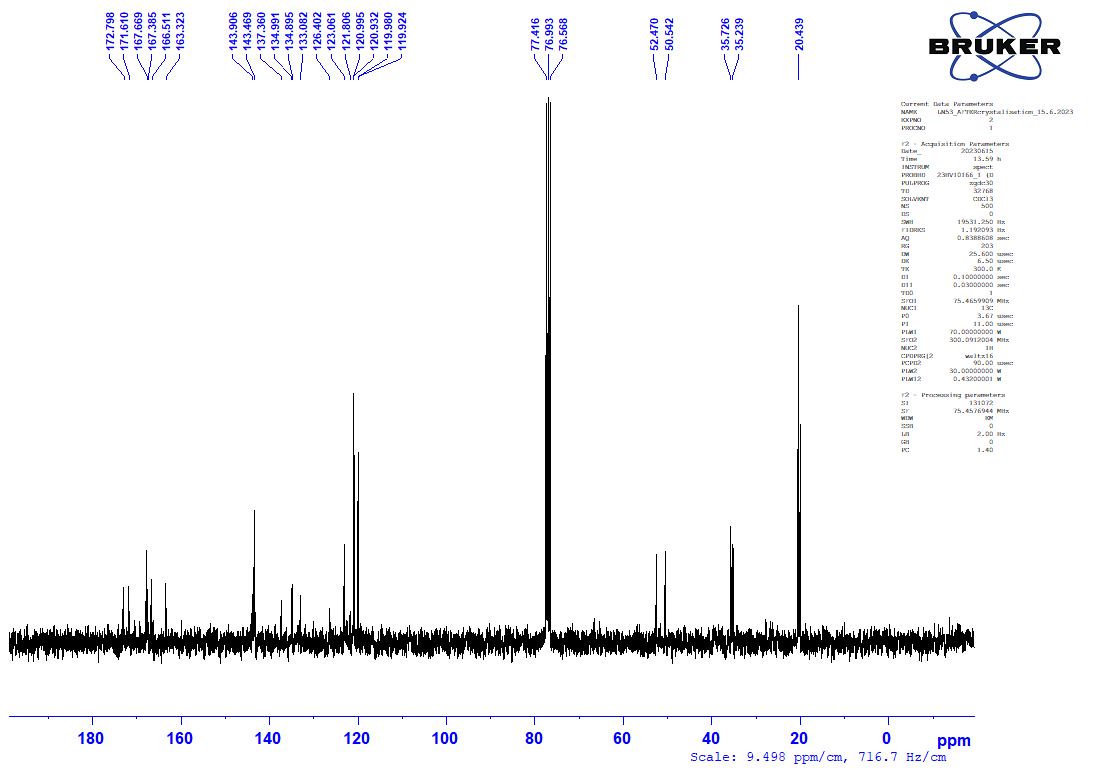


**Figure S1b.** ¹³C NMR spectrum of LN-53 (CDCl₃, 75 MHz).


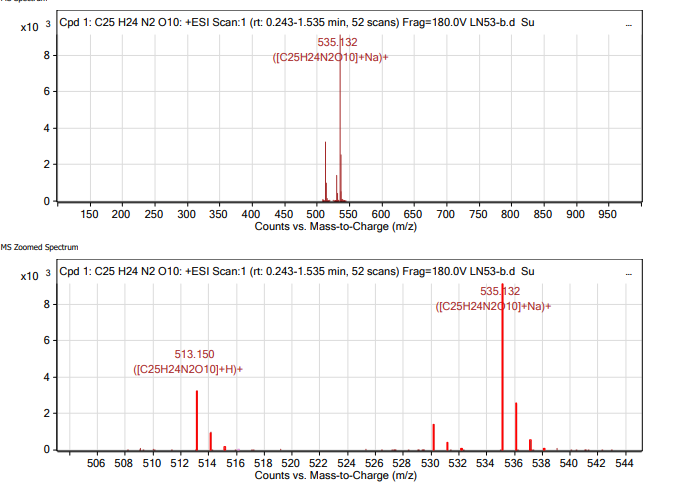

**Figure S1c.** mass spectroscopy of LN-53.
**HRMS (ESI⁺):** *m/z* calcd for C₂₅H₂₅N₂O₁₀⁺ [M+H]⁺: 513.1501; found: 513.1498

**Uncut Western Blot Images**

Western Blot Images for 1-h treatment


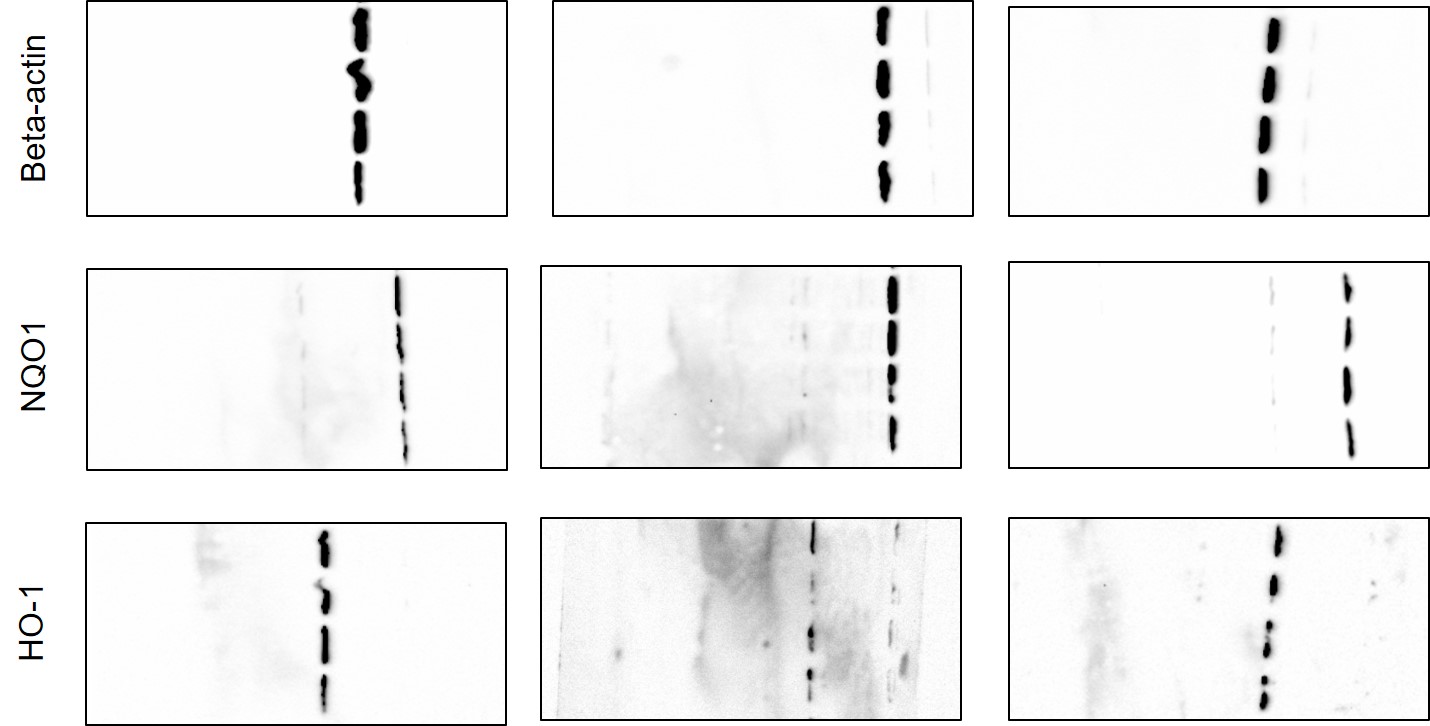


**Figure S2a**. Uncut Western Blot Images of 1-hour treatment.

Western Blot Images for 3-h treatment


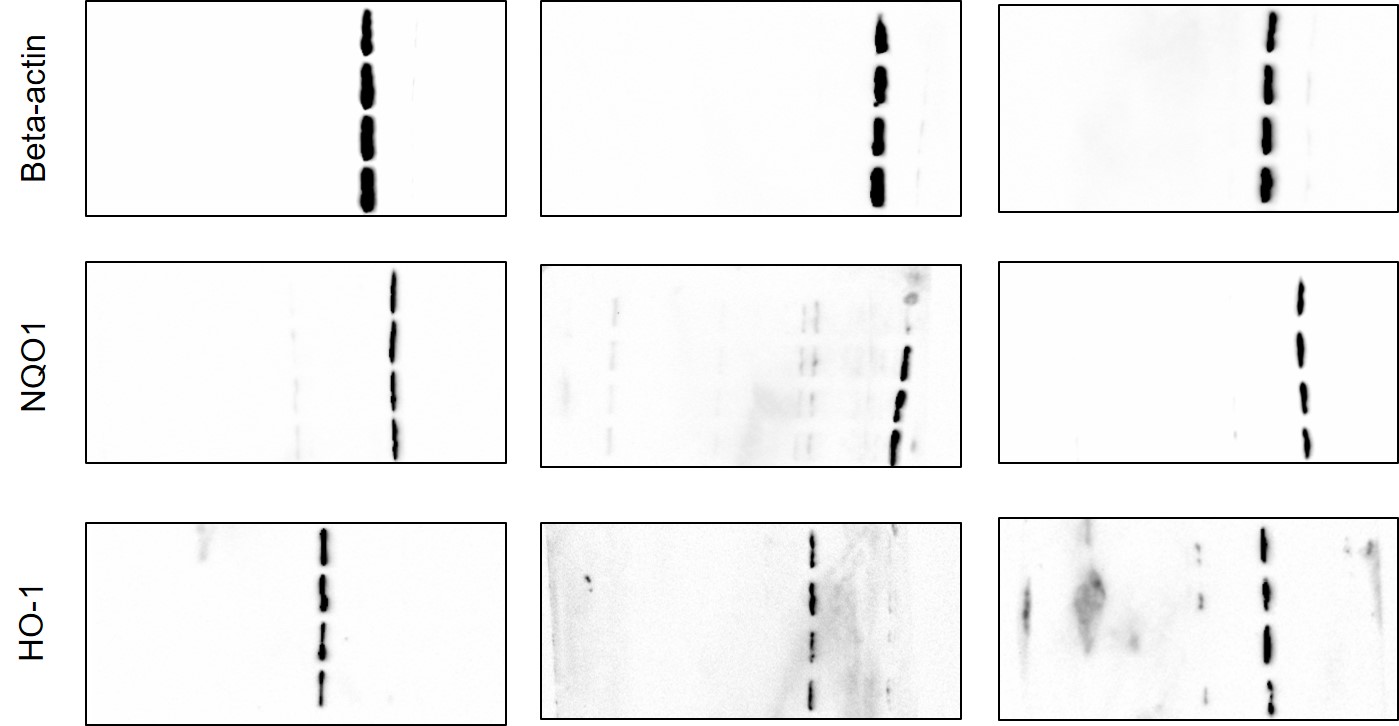


**Figure S2b.** Uncut Western Blot Images of 3-hour treatment.

**Statistics details**

***Table S1. The details of statistics performed in Figure 2***

| **Assay** | **Detail** | **Figure** | **F (DFn, DFd)** | **p-value of the test** |
| --- | --- | --- | --- | --- |
| **MTT** | 1-h MTT | Fig. 2B | F_7, 16_=2.601 | 0.0537 |
|  | 3-h MTT | Fig. 2E | F_7, 16_=0.3062 | 0.9407 |
|  | 6-h MTT | Fig. 2H | F_7, 16_=5.584 | 0.0021 |
|  | 24-h MTT | Fig. 2K | F_7, 16_=87.11 | <0.0001 |
| **LDH** | 1-h LDH | Fig. 2C | F_8, 18_=3247 | <0.0001 |
|  | 3-h LDH | Fig. 2F | F_8, 18_=2406 | <0.0001 |
|  | 6-h LDH | Fig. 2I | F_8, 18_=3235 | <0.0001 |
|  | 24-h LDH | Fig. 2L | F_8, 18_=3214 | <0.0001 |
| **Caspase Assay** | Caspase-3 | Fig. 2M | F_7, 16_=19.14 | <0.0001 |
|  | Caspase-8 | Fig. 2N | F_7, 16_=117.6 | <0.0001 |
|  | Caspase-9 | Fig. 2O | F_7, 16_=1020 | <0.0001 |

Test: Ordinary one-way ANOVA

***Table S2. The details of statistics performed in Figure 3C***

| ***Comparison*** | ***1-h*** | ***2-h*** | ***3-h*** | ***4-h*** | ***5-h*** | ***6-h*** | ***12-h*** | ***24-h*** |
| --- | --- | --- | --- | --- | --- | --- | --- | --- |
| ***US vs TBHP*** | *<0.0001* | *<0.0001* | *<0.0001* | *<0.0001* | *<0.0001* | *<0.0001* | *<0.0001* | *<0.0001* |
| ***TBHP vs TBHP+1 μM*** | *0.0032* | *0.0003* | *0.0027* | *0.0409* | *0.0092* | *0.0527 (ns)* | *0.0097* | *0.0462* |
| ***TBHP vs TBHP+2.5 μM*** | *0.0055* | *0.0004* | *0.0012* | *0.0214* | *0.0037* | *0.2623 (ns)* | *0.0334* | *0.1670 (ns)* |
| ***TBHP vs TBHP+5 μM*** | *<0.0001* | *<0.0001* | *<0.0001* | *<0.0001* | *<0.0001* | *<0.0001* | *<0.0001* | *<0.0001* |
| ***TBHP vs TBHP+10 μM*** | *<0.0001* | *<0.0001* | *<0.0001* | *<0.0001* | *<0.0001* | *<0.0001* | *<0.0001* | *0.0281* |

Test: Ordinary two-way ANOVA

| Interaction | F_63, 160_=1.344 | p=0.0718 |
| --- | --- | --- |
| Row Factor | F_9, 160_=404.5 | p<0,0001 |
| Column Factor | F_7, 160_=15.61 | p<0,0001 |
